# The Local Regulation of Vascular Function: From an Inside-Outside to an Outside-Inside Model

**DOI:** 10.3389/fphys.2019.00729

**Published:** 2019-06-12

**Authors:** Eduardo Nava, Silvia Llorens

**Affiliations:** Department of Medical Sciences, Faculty of Medicine of Albacete, Centro Regional de Investigaciones Biomédicas (CRIB), University of Castilla-La Mancha, Albacete, Spain

**Keywords:** prostaglandins, endothelium-derived NO, PVAT-derived NO, EDHF, EDCF, adventitia, perivascular adipose tissue

## Abstract

Our understanding of the regulation of vascular function, specifically that of vasomotion, has evolved dramatically over the past few decades. The classic conception of a vascular system solely regulated by circulating hormones and sympathetic innervation gave way to a vision of a local regulation. Initially by the so-called, autacoids like prostacyclin, which represented the first endothelium-derived paracrine regulator of smooth muscle. This was the prelude of the EDRF-nitric oxide age that has occupied vascular scientists for nearly 30 years. Endothelial cells revealed to have the ability to generate numerous mediators besides prostacyclin and nitric oxide (NO). The need to classify these substances led to the coining of the terms: endothelium-derived relaxing, hyperpolarizing and contracting factors, which included various prostaglandins, thromboxane A2, endothelin, as well numerous candidates for the hyperpolarizing factor. The opposite layer of the vascular wall, the adventitia, eventually and for a quite short period of time, enjoyed the attention of some vascular physiologists. Adventitial fibroblasts were recognized as paracrine cells to the smooth muscle because of their ability to produce some substances such as superoxide. Remarkably, this took place before our awareness of the functional potential of another adventitial cell, the adipocyte. Possibly, because the perivascular adipose tissue (PVAT) was systematically removed during the experiments as considered a non-vascular artifact tissue, it took quite long to be considered a major source of paracrine substances. These are now being integrated in the vast pool of mediators synthesized by adipocytes, known as adipokines. They include hormones involved in metabolic regulation, like leptin or adiponectin; classic vascular mediators like NO, angiotensin II or catecholamines; and inflammatory mediators or adipocytokines. The first substance studied was an anti-contractile factor named adipose-derived relaxing factor of uncertain chemical nature but possibly, some of the relaxing mediators mentioned above are behind this factor. This manuscript intends to review the vascular regulation from the point of view of the paracrine control exerted by the cells present in the vascular environment, namely, endothelial, adventitial, adipocyte and vascular stromal cells.

## Introduction

Early physiologists ascribed the regulation of the vascular tone to hormones released by specific endocrine glands or to the autonomous nervous system. A singular breakthrough against this notion was the proposal made in the mid of last century by Spanish physicians who observed the blood pressure effects of arterial extract injections. They proposed the existence of an endocrine function within the vascular wall. The hormonal functions were not yet assigned to specific vascular structures but were considered endocrine, rather than paracrine (Jimenez Diaz et al., 1954). The notion of paracrine secretion was introduced shortly after for its convenience in distinguishing gastrointestinal hormones acting locally from hormones with systemic action (Feyrter, 1952). However, the term “paracrine” took quite a long time to become of common use.

It is of justice to admit that before the unprecedented advance made with the introduction of endothelium-derived relaxing factor (EDRF) (Furchgott and Zawadzki, 1980), later identified as nitric oxide (NO), the discovery of prostacyclin should be recognized as the earliest approach to a tissue-specific release of a vasomotor mediator from and toward the arterial wall itself (Moncada et al., 1976, 1978). The notion of endothelial cells releasing substances that preferentially affect smooth muscle cells, which is the sense of paracrine control, was extended during the eighties to other substances, such as NO, prostanoids or endothelin. Under this point of view, the endothelial cell was designated as a full endocrine organ (Vane et al., 1987). Over the years, other vascular cells were included as paracrine regulatory cells. Incongruous as it may seem, the first non-endothelial vascular tissue to qualify as paracrine is the distant perivascular fat tissue (Soltis and Cassis, 1991) rather that the closer adventitial fibroblast. However, these precocious scientists could not see the surge of research on perivascular fat until 2002, when the first adipocyte-derived vasoactive factor was described (Löhn et al., 2002). In the meantime, new roles were assigned to adventitial cells as a source of paracrine substances (Pagano et al., 1995) and a new concept in the control of vasomotion based on an outside-to-inside regulation surged.

**FIGURE 1:**
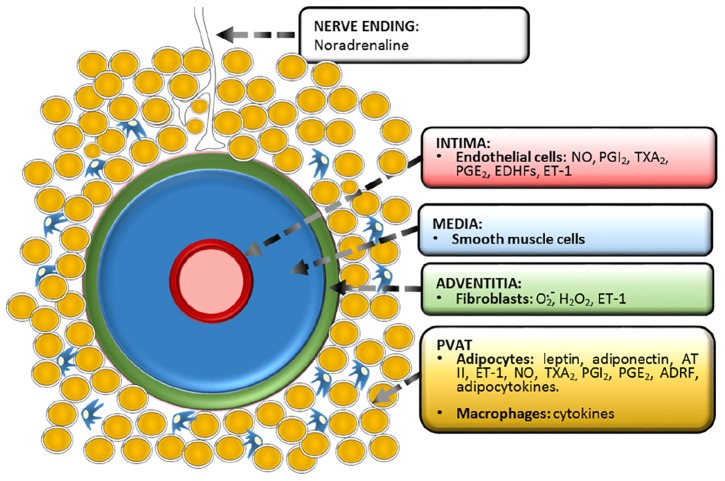
Functional anatomy of a vessel showing the three classic *tunicae* plus the fourth proposed layer, PVAT or *tunica adiposa*. The paracrine substances produced by each layer are depicted. Prostacyclin (PGI_2_), thromboxane A_2_ (TXA_2_), prostaglandin E_2_ (PGE_2_), endothelium-derived hyperpolarizing factors (EDHFs), endothelin-1 (ET-1), angiotensin II (AT II), adipose-derived relaxing factor (ADRF).

This review is centered in the paracrine vascular self-regulation of its own diameter. We have left aside the hormonal and nervous regulation as well as gasotransmission by molecules other than NO, which has been extensively reviewed (Cebova et al., 2016; Dongo et al., 2018). Is important to stress that it is not always easy to establish when a mediator is solely paracrine or has endocrine properties as well. Figure 1 shows the vessel’s layers and the paracrine substances produced by each one and Table 1 displays in chronological order the major events of vascular paracrine research and discovery.

**Table 1:** Major events of vascular paracrine finding chronologically ordered.

Event	References
Discovery of a vasodilatory prostaglandin synthesized and released by the vessel wall	Moncada et al., 1976
Chemical structure and coining of the term: prostacyclin	Whittaker et al., 1976
The endothelium as the most abundant source of prostacyclin	Moncada et al., 1977
Activation of guanylate cyclase by NO and nitrocompounds	Katsuki and Murad, 1977
Discovery of the role of endothelium as a source of vasodilating substances upon stimulation	Furchgott and Zawadzki, 1980
Earliest confirming reports	Altura and Chand, 1981; De Mey and Vanhoutte, 1981b
Endothelium-dependent vasoconstriction	De Mey and Vanhoutte, 1981a
First use of the acronym: EDRF	Cherry et al., 1982
First use of the term: nitrovasodilators	Rapoport et al., 1982
Endothelium-dependent hyperpolarization of smooth muscle	Bolton et al., 1984
Existence of an endothelial cell-derived vasoconstrictor substance of polypeptidic nature	Hickey et al., 1985
Evidence of endothelial release of a diffusible vasoconstrictor substance	Rubanyi and Vanhoutte, 1985
Endothelium-dependent contractions are mediated by endothelial prostaglandins	Miller and Vanhoutte, 1985
Incorporation of the acronym: EDCF	Gillespie et al., 1986
Chemical nature of EDRF as NO	Palmer et al., 1987
Earliest confirming reports	Khan and Furchgott, 1987; Ignarro et al., 1987
Identification of L-arginine as the precursor of NO	Palmer et al., 1988
Isolation of endothelin	Yanagisawa et al., 1988
Incorporation of the term: EDHF	Weston et al., 1988
Endothelial release of a diffusible hyperpolarizing substance	Feletou and Vanhoutte, 1988
Influence of perivascular adipose tissue on smooth muscle responsiveness	Soltis and Cassis, 1991
Assignment of a paracrine role to adventitial cells	Di Wang et al., 1999
Adventitium-derived relaxing factor coined as ADRF	Löhn et al., 2002
Demonstration that ADRF hyperpolarizes smooth muscle cells	Verlohren et al., 2004
Incorporation of the acronym: PVAT	Gao et al., 2005a
Existence of a procontractile transferable substance from PVAT	Gao et al., 2006
*Tunica adiposa* as the fourth layer of the vascular wall histologically acknowledged	Chaldakov et al., 2007
Incorporation of the term: perivascular adipocyte-derived constricting factor PVCF	Gao, 2007
Incorporation of the term: adipocyte-derived hyperpolarizing factor ADHF	Weston et al., 2013


## The Endothelial Inside-To-Outside Conception

Although endothelial regulation is today profoundly integrated in biomedical culture, this has not always been so. During most of last century, the intimal endothelium was considered a mere sort of tapestry with no physiological activity. Electron microscopist, Florey referred to these cells as “*nucleated cellophane*” cells (Florey., 1966). It is of justice to mention, however, that one of the early electron microscope pioneers, Rhodin (1967), who also observed the endothelium, intuitively suggested that transmitter substances present in the blood might be conveyed to the luminal side of endothelial cells and forwarded to smooth muscle cells. Although EDRF, later identified as NO, has gained the attribute of the canonical substance derived from the endothelial cells, it must be emphasized that, strictly speaking, the discovery of prostacyclin by Vane’s group (Moncada et al., 1976) represented the turning point between Florey’s useless “cellophane-like” cells and a fully functional paracrine cell. The report on the newly discovered prostacyclin (which they still referred to as PGX) emphasized that is locally produced within the arterial wall and should play a role in the control of local vascular tone (Moncada et al., 1976). They did not pinpoint any specific part of the vessel responsible for the formation of this autacoid (the term used to refer to these local mediators). A forthcoming publication dealt with the ability displayed by the different tunicae of the vessel wall to release prostacyclin. This showed that prostacyclin was highest in the endothelial layer, thus endowing the endothelium a role in the local control of vascular tone (Moncada et al., 1977).

Prostacyclin production was regarded for its anti-aggregating properties and endothelial impairment was considered as a problem of hemostasis, rather than a vasomotor one. This scenario radically changed in 1980 when Furchgott and Zawadzki (1980) demonstrated that endothelial cells play an obligatory role in the relaxation of arterial smooth muscle when stimulated by acetylcholine and introduced the term *endothelium-derived relaxing factor* and its acronym *EDRF* (Cherry et al., 1982). EDRF was identified as NO in 1987 by Palmer et al. (1987), and immediately confirmed by Khan and Furchgott (1987), as well as Ignarro et al. (1987).

An independent confirmation of the obligatory roles of the endothelium in acetylcholine relaxation after Furchgott’s seminal paper was reported by De Mey and Vanhoutte (1981b), who also initiated the discovery of the “saga” of substances that vasodilate in an endothelium-dependent manner. They also showed that the endothelium is a source of substances that mediate contractile reactivity of the smooth muscle via the discharge of diffusible material acting just in an opposite manner to that of EDRF (Rubanyi and Vanhoutte, 1985).

Rubanyi together with Hickey, using a setup intended to uncover the chemistry of Furchgott’s factor, found a peptidic transferable substance that actually had contracting properties (Hickey et al., 1985). This was later characterized and named endothelin (Yanagisawa et al., 1988). Gillespie proposed to use the acronym EDCF to distinguish it from EDRF (Gillespie et al., 1986).

**FIGURE 2:**
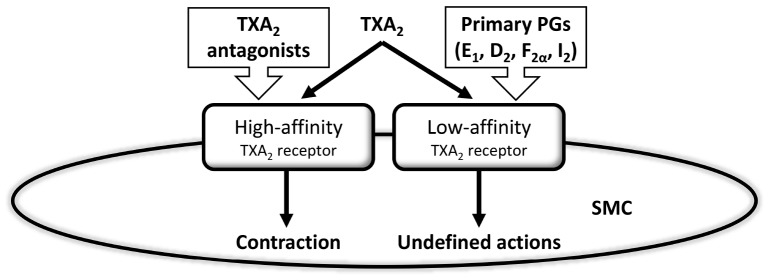
Hanasaki and Arita’s early vision of prostaglandin’s action on smooth muscle cell receptors was a prelude of much research on the role of different prostaglandins, and most especially prostacyclin, acting as a vasoconstrictor and, thereby, functioning as an endothelium-derived contracting factor. Thromboxane A2 (TXA_2_), prostaglandins (PGs), smooth muscle cell (SMC). Modified from Hanasaki and Arita (1989).

Vanhoutte found difficult to assume that endothelin was the only substance behind endothelium-dependent contractions (Vanhoutte et al., 1989). A previous paper actually showed that endothelial cyclooxygenase generates vasoconstrictor prostaglandins (Miller and Vanhoutte, 1985), which were specifically ascribed to TXA_2_ (Altiere et al., 1986). Hanasaki and Arita (1989), in the late eighties, demonstrated that smooth muscle cells possess common binding sites for TXA_2_ and various other prostaglandins, including PGI_2_, which worked as high affinity receptors for TXA_2_ and as low affinity for the rest of prostaglandins (Figure 2). They proposed that these common receptors may account for the contraction elicited by TXA_2_, but did not discuss the possible effects of other prostaglandins when binding to the low affinity site of the TXA_2_ receptor. This paper, however, inspired the idea that, in some cases, PGI_2_ could preferentially activate common-prostaglandin TXA_2_ receptors rather than the natural PGI_2_ receptors. This was taken to the physiological arena by Rapoport’s group (Williams et al., 1994). The “multiprostaglandin” TXA_2_ receptor shown by Hanasaki and Arita was the basis for the proposal that PGI_2_ serves as the mediator underlying EDCF (Rapoport and Williams, 1996).

At some point, it became obvious that EDRF (later NO) and prostacyclin were not enough to account for all the relaxing attributes of the vessels (De Mey et al., 1982). Another evidence that did not fit in the endothelium-derived vasoactive release scheme was the long known observation that acetylcholine-mediated vasodilation was detectable, not solely on the neighboring regions, but also lengthwise the vessel wall exceeding the notion of “paracrinity” (Hilton, 1959). There had to be another unknown mechanism. Bolton et al. (1984) forwarded the possible presence of a substance produced by endothelial cells, which causes hyperpolarization. This was coined with the term “endothelium-derived hyperpolarizing factor” (EDHF) (Weston et al., 1988). Feletou and Vanhoutte (1988) carried out experiments aimed to bioassay the substance(s) causing hyperpolarization and provided evidence of the diffusible nature of EDHF. In 1998, Edwards et al. (1998) demonstrated that hyperpolarization could be due to K^+^ coming from the endothelial cell. There was indeed a K^+^ efflux from the endothelium secondary to a cholinergic activation. This cationic escape was demonstrated to be through endothelial calcium-dependent K^+^ channels. It was also shown that this cation, once present in the extracellular liquid, elicits hyperpolarization through inwardly rectifying K^+^ channels and the activation of a Na^+^/K^+^-ATPase.

As we have seen, the problem posed by some on the necessity of a third vasodilating factor (NO and prostacyclin being the other ones) to explain all sorts of relaxing phenomena, had been successfully addressed with EDHF. However, the “remote” acetylcholine vascular relaxation described by Hilton (1959), had no satisfactory answer on the basis of EDHF. It had to be admitted that the change in membrane potential could be transmitted electrotonically through discrete connecting structures in the membranes named myoendothelial gap junctions (Spagnoli et al., 1982). The spread of hyperpolarization has been studied in depth by Segal and Duling’s group (Segal, 2015). Therefore, the problem of phenomena difficult to reconcile with the endothelium-derived vasoactive release scheme transmission was solved. Moreover, with myo-endothelial gap junctions in the scene, an EDHF as a paracrine mediator is not necessary after all and the term EDH, rather than EDHF, is currently recommended (Feletou and Vanhoutte, 2013).

## Beyond the Smooth Muscle Wall: New Paracrine Cells

For decades, the leader of the paracrine control of the vessel’s function was the luminal endothelium, and NO the main character. The other side of the vessel, the abluminal layer had received very little attention. Some histologists and pathologists claim that the *tunica adiposa* should be considered together with the classic *tunica intima, tunica media* and *tunica adventitia* (Chaldakov et al., 2007, 2012). Interestingly, this claim has a functional background rather than a histological one. In this way, perivascular adipose tissue (PVAT), which had always been considered as “extravascular,” is increasingly considered an “intravascular” structure fundamentally composed by adipocytes, and also macrophages, fibroblasts, dendritic cells, pericytes, endothelial cells, and nerve endings (Stenmark et al., 2013).

## The Paracrine Roles of Adventitial Fibroblasts

One of the reasons why the adventitia had been persistently overlooked is the fact that it is technically very hard to remove (Kemler et al., 1997). Thus, it is far more difficult to investigate its roles in the same way as with the endothelium, in which removal is fairly easy. Most frequently, the *tunica adventitia* has been considered simply as a passive scaffolding of the vessel. Possibly, the study carried out by Pagano et al. (1995) was the earliest to propose an active role of the adventitia in the regulation of vascular function by showing the existence of a powerful superoxide-generating system within this layer. Shortly before Pagano’s introductory paper (Pagano et al., 1995), another suggestive study was published. This one emphasized that the adventitia could work as functional barrier against endothelial NO (Steinhorn et al., 1994). The study carried out by Pagano on the production of superoxide by the adventitia was followed by a number of clarifying reports. It was shown that the superoxide generating enzyme, NADPH oxidase is located within the fibroblasts of the *tunica adventitia* (Pagano et al., 1997). The barrier identified by Steinhorn was ascribed to superoxide produced by fibroblasts, which would inactivate NO and therefore act as a chemical barrier (Wang et al., 1998). In 1999, adventitial superoxide was “officially” established as a paracrine player in the control of the vessel wall (Di Wang et al., 1999). In this way, the fibroblast of the adventitia could be considered a paracrine cell in the control of vasomotion.

It is obvious that to carry out experiments aimed to understand how the adventitia contributes to the regulation of vascular tone it is important to be able to remove it properly. The technical problems to obtain vessels lacking adventitial cells have been solved using different procedures. For example, Gonzalez et al. (2001) combined physical removal with a collagenase treatment. They were able to verify that when the *tunica adventitia* was present, smooth muscle contractions were stronger and endothelium-dependent relaxations weaker. These experiments unmistakably proved that the adventitial layer had a role in the regulation of vasomotion (Gonzalez et al., 2001). In this case, again superoxide (Rey et al., 2002), and more recently hydrogen peroxide (Cascino et al., 2011) were considered responsible for the influences the adventitia has on endothelial function. Additionally, fibroblasts have been shown to produce endothelin-1. Therefore, this peptide can be added to the relatively short list of fibroblast-derived paracrine substances (An et al., 2006).

## Perivascular Adipose Tissue, a New Era in Vascular Research

In contrast to the adventitia, PVAT offers little resistance to removal. Ironically, this caused that the vasoactive roles of perivascular fat were always missed. On the assumption that PVAT is a extraneous tissue to the vessel, it has been always removed on a routine basis. Worse, it has been considered a diffusion barrier against vasoactive substances aimed at “authentic” vascular tissue. This situation has now changed and we are witnessing a rush for PVAT research. However, it must be said that it has been known for years that adipocytes are able to produce hormones involved in the control of energy balance, the so-called, *adipokines* (Hotamisligil et al., 1993). What was really new in the vascular field was that adipocytes might work as paracrine cells by releasing vasomotor transferable mediators. Evidently, this could only take place when adipose tissue is next to the vessel wall, something that happens only with adventitial fat or PVAT (Figure 1). In this case, adipocytes would co-regulate smooth muscle tone together with intimal endothelial cells. By demonstrating that the fat tissue around the adventitia releases a transferable substance that alters the vessel tone, this novel idea was materialized. In 2002, Löhn et al. (2002) demonstrated for the first time the existence of a diffusible substance of PVAT origin, which they called ADRF, the acronym of “*adventitium-derived relaxing factor*,” a name that strongly recalls that of EDRF (Cherry et al., 1982, Table 1). In an ensuing publication, the “A” of ADRF was for “adipocyte” rather than “adventitium” (Verlohren et al., 2004), thus emphasizing the role of adipose tissue over conventional adventitial cells. In the earliest publication of the group it was shown that the contractile force elicited by aortic rings upon phenylephrine, angiotensin II or serotonin, assessed by cummulative dose-response curves, was systematically weaker when the vessel was examined with its perivascular fat preserved than when it was removed. It was also demonstrated that a solution obtained from PVAT-incubated specimens caused relaxations when supplied to vessels completely devoid of fat and precontracted with any of the mentioned vasoconstrictors (Löhn et al., 2002; Verlohren et al., 2004).

The idea of a functional perivascular adipocyte has generated a novel manner in which we regard the vessel, both anatomical (regarding PVAT as the fourth layer of the vascular wall), physiological (perivascular adipocytes acting together with the endothelial cell) and even pathophysiological: the so-called “perivascular adipose tissue dysfunction” (Guzik et al., 2007). Even more, the *adventitia/media thickness* factor (which includes PVAT) rather than the conventional parameter *intima/media thickness* has been recommended for ultrasonographic measurements since the former is regarded more precise than the latter in relation to cardiovascular risk (Skilton et al., 2009).

Although PVAT is an attractive alternative to explain the paracrine functions of the vessel, there are substantial dissimilarities between perivascular adipocytes and endothelial cells. Thus, it would be naïve to consider adventitial fat as the *new endothelium*. Namely, these differences are: (a) the most evident, but most important, is that endothelial cells are exposed to the effect of the blood stream and this determines the existence of a direct sensing of the mechanical conditions (pressure and shear stress) of the blood. PVAT, in contrast, is more exposed to tissue inflammatory conditions. (b) Provided there is no injury, the endothelium is always present. By contrast, PVAT is missing in a vast portion of the circulatory system: all the capillary circulation (with the exception of that serving adipose tissue) lacks adipose tissue. (c) Adipose tissue normally surrounds large conduit arteries and veins as they run through adiposity, however, fat is usually left behind once the vessel comes into or leaves the target organ, a fact that obviously does not happen with endothelium. (d) While PVAT is made out of many cell types, the intimal layer is made solely by endothelial cells and subendothelial fibroblasts. Conversely, both tissues share some aspects. For example, the phenotype of PVAT is different depending on the site of the fat store (Chatterjee et al., 2009; Rittig et al., 2012; Padilla et al., 2013; Owen et al., 2014) and this affects the way it regulates the tone of the surrounding vessel (Gil-Ortega et al., 2015). This evokes the heterogeneous ability of generating different endothelium-dependent dilating or contracting substances displayed by the endothelium depending on the vascular region (De Mey and Vanhoutte, 1982).

**FIGURE 3:**
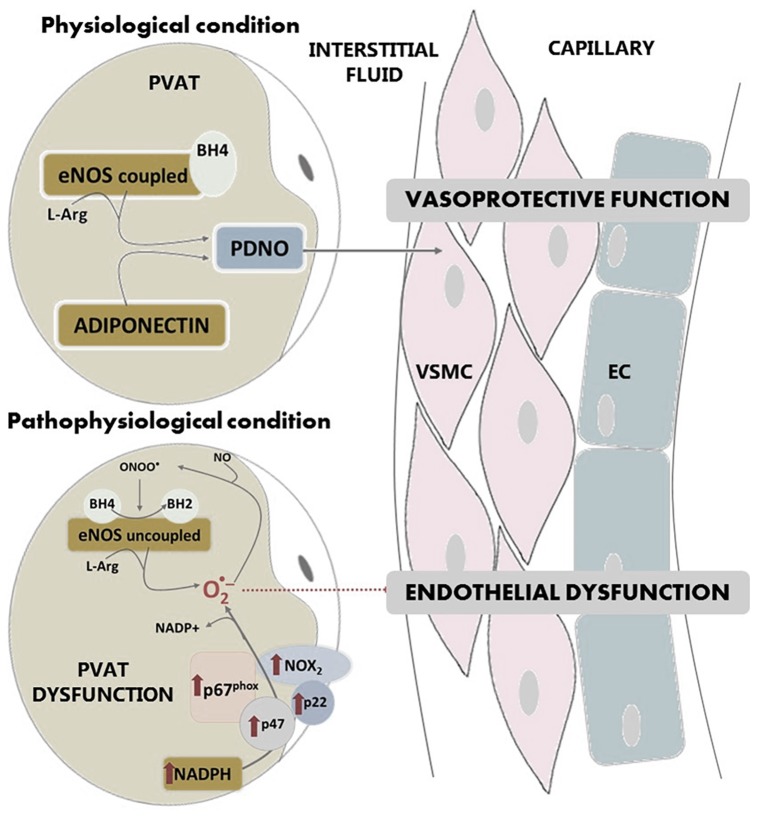
In physiological conditions, eNOS is expressed in PVAT and produces NO. PVAT-derived NO diffuses into the capillaries protecting endothelial function. In pathological conditions, the production of PDNO is compromised due to superoxide overproduction in PVAT, which is formed by NADPH oxidase and uncoupled eNOS. Activity of NADPH oxidase is increased in this tissue. Overproduction of superoxide anion by uncoupled eNOS or NADPH oxidase leads to peroxynitrite formation, which in turn produces oxidation of BH4 to BH2, an essential cofactor of eNOS. PVAT: perivascular adipose tissue, VSMC: vascular smooth muscle cells; EC: endothelial cells; eNOS: endothelial isoform of nitric oxide synthase; NO: nitric oxide; PDNO: PVAT-derived NO; ONOO^-^: peroxinitrite; O_2_^-^: superoxide anion; L-Arg: L-Arginine; BH4: tetrahydrobiopterin; BH2: dihydrobiopterin; p67^phox^, p47 and p22 and NOX2: NADPH oxidase subunits.

Another common attribute of PVAT and the endothelium is the capability to synthesize NO and prostaglandins (Dashwood et al., 2007; Chang et al., 2012). Regarding NO, various groups have detected the expression of the endothelial isoform of NO synthase (eNOS) in the PVAT surrounding the thoracic aorta (Araujo et al., 2015; Xia et al., 2016; Victorio et al., 2016) as well as the ability of this tissue to release NO. This PVAT-derived NO (PDNO) is released into the interstitial fluid and diffuses into the capillaries causing vasodilatation (Mastronardi et al., 2002), which is key in the vasoprotective function of the endothelium (Figure 3). Victorio and coworkers demonstrated that PVAT presents heterogeneity in its eNOS expression according to the vascular region studied: thoracic or abdominal aorta (Victorio et al., 2016). In contrast, endothelial eNOS expression remains the same along the aorta.

As for the other isoforms of NO synthase, the inducible isoform (iNOS) has been identified under basal conditions in very low levels in white adipose tissue (Ribiere et al., 1996; Elizalde et al., 2000) but clearly induced after LPS treatment (Ribiere et al., 1996). Neuronal NO synthase (nNOS) was not found in Elizalde’s study (Elizalde et al., 2000), but, in a study specifically focused in PVAT of mouse aorta, mRNA expression of the three isoforms of NO synthase, eNOS, nNOS and iNOS was detected (Xia et al., 2016).

In pathological conditions, the production of PDNO may be compromised due to superoxide overproduction in PVAT. This tissue, together with other vessel wall components (endothelium, smooth muscle and adventitia) is a source of vascular superoxide. This production of superoxide anion leads to oxidative stress, which is an important part of endothelial dysfunction. Although NADPH oxidase is the main superoxide-producing enzyme, the uncoupling of eNOS also produces this anion. It has been shown that under pathological conditions, NADPH oxidase activity in PVAT is increased (Marchesi et al., 2009), as well as the expression of the subunits of the NADPH oxidase complex such as p67phox, p22phox, p47phox or Nox2 (Gao et al., 2006; DeVallance et al., 2018; Quesada et al., 2018). In addition, PVAT displays an increased mRNA expression of the Ncf2 gene that encodes for the p67phox subunit (Ketonen et al., 2010). All this contributing to vascular superoxide production and endothelial dysfunction (Figure 3).

Uncoupled eNOS (Figure 3) no longer produces NO, but superoxide. NOS uncoupling has been demonstrated in numerous conditions and tissues, of which PVAT is not an exception (Xia et al., 2016). Uncoupled eNOS considerably contributes to endothelial dysfunction, since it not only reduces NO production, but also potentiates the preexisting oxidative stress. The situation is further aggravated because the overproduction of superoxide anion by uncoupled eNOS leads to peroxynitrite formation, that in turn depletes tetrahydrobiopterin (BH4), which is an essential cofactor of eNOS. In PVAT, oxidation of BH4 to BH2 (dihydrobiopterin), is likely to be a major cause of eNOS uncoupling and endothelial dysfunction (Xia et al., 2016).

More that a decade before Löhn’s publication, Soltis and Cassis published the earliest report suggesting that PVAT influences the vessel’s contractility (Soltis and Cassis, 1991). Interestingly, they used the term “perivascular adipose tissue” for this early paper, a term that has been adopted to refer to the *tunica adiposa*. Perhaps, because of this this, Soltis and Cassis’ work has been occasionally misunderstood and quoted as the first one to demonstrate the existence of a prorelaxing function of PVAT. In fact, what Soltis and Cassis show is PVAT as the physical support of neurotransmitter reuptake by sympathetic fibers. These endings can be found in PVAT and in the adventitia (Figure 1). These two researchers noted that, in the absence of PVAT, there was an abolishment of noradrenaline-mediated contractions. The sensitivity to noradrenaline was also decreased when arterial rings had a preserved PVAT. They showed that this is produced by the reuptake of noradrenaline by PVAT and suggested that this is carried out by the nerve endings within PVAT present in the synaptic gap. This causes a weaker effect of the drug. However, at no time they asserted that PVAT produces any kind of anticontractile or relaxing substance. Actually, their experiment was only possible to carry out with noradrenaline, which suffered reuptake. It did not work with phenylephrine or potassium, which did not undergo reuptake. Much more recently, the interaction between sympathetic innervation, PVAT and smooth muscle relaxation has received further attention in a work showing that PVAT adipocytes can transport and store noradrenaline (Ayala-Lopez et al., 2015) and that this acts as a reservoir of the sympathetic nerve ending release of the catechoamine, therefore preventing smooth muscle contraction (Saxton et al., 2018).

## The Nature and Physiological Properties of PVAT-Derived Factors

The substances produced by adipocytes are collectively known as adipokines (Trayhurn and Wood, 2004). Today there are more than one hundred established adipokines (Halberg et al., 2008). Considering that adipocytes represent more than sixty percent of the adiposity and their enormous hormone-synthesizing capability, it is not unreasonable to believe that released adipokines from adipocytes located in the vicinity of the artery, i.e., PVAT, travel to the *tunica media*, or even the *intima*, and alter, in some way, vascular function (Figure 1). Today, this paracrine crosstalk between PVAT and its artery, or “vasocrine” communication (Yudkin et al., 2005) is fully recognized by the scientific community (Nava and Llorens, 2016). Amid all the recorded adipokines, many have vasoactive power to a certain degree apart from their central function. From the point of view of the vascular physiologist, adipokines can be classified as follows:

(a) Unidentified substances from the perivascular environment
(b) Adipocyte-derived vasoactive hormones
(c) Energy balance adipokines
(d) Free radicals
(e) Adipocytokines

It is also possible to organize vasoactive adipokines on the basis of the differential action on smooth muscle (direct pro- or anticontractile effect) or on endothelial cells (stimulating or inhibiting the release of endothelium-derived vasoactive substances).

### (a) Unidentified Substances From the Perivascular Environment

These have been solely investigated in PVAT and in no other fat depot. The experiments that led to the knowledge of the presence of this kind of substances basically consist in the transfer of a physiological buffer from isolated arteries in which PVAT had been removed (Malinowski et al., 2008) or preserved (Löhn et al., 2002) to another artery devoid of PVAT. Alternatively, dose-response curves of a vasoconstrictor are carried out in segments with, versus without, PVAT. It this case, more powerful constrictions take place when PVAT is removed. Consequently, these unidentified PVAT-borne substances are sometimes referred to as anti-contractile substances, but more frequently as ADRFs. They are probably released by PVAT and no other fat store but this point has never been studied so far.

Initial reports on rat aorta show that ADRFs relax the vessel via hyperpolarization by opening different kinds of potassium channels on the membrane of the smooth muscle (Löhn et al., 2002; Verlohren et al., 2004). More specifically, K^+^_ATP_ channels have been shown to be involved and opening appears to be dependent on extracellullar calcium, protein kinase A and tyrosine kinase, but independent of the presence of the endothelium or perivascular nerves (Löhn et al., 2002; Dubrovska et al., 2004). In the rat mesenteric artery, K^+^_V_ delayed rectifier channels were shown to hyperpolarize the smooth muscle cell membrane when opened by the transferable substance (Verlohren et al., 2004). On the other hand, in human arteries, K^+^_Ca_ channels are the target of ADRF (Gao et al., 2005b). However, the same researchers report that PVAT does not diminish contractions of rat mesenteric arteries when elicited by electrical stimulation (Gao et al., 2006). They reasoned that this is specific to PVAT’s perivascular nerve endings where NAD(P)H oxidase synthesizes superoxide anion and, as a result of this, PVAT mediates contraction instead of relaxation (Gao et al., 2006). In a later review, they suggested to use the term *perivascular adipocyte-derived constricting factor* (PVCF) for these type of contractile substances (Gao, 2007). More recently, Meyer et al. (2013) have used the acronym ADCF. It was suggested that substances different from oxygen radicals could act as PVCFs. In this sense, PVAT might have a double role on the vessel surrounded: anti-contractile and pro-contractile (Gao, 2007). Research carried out later uncovered new substances acting as PVCFs after electrical stimulation, like angiotensin II (Lu et al., 2010).

Further experimentation focused on anti-contractile transferable substances have shown that the factor released by rat aortic PVAT works through two mechanisms (Gao et al., 2007). One of the mechanisms is endothelium-independent but, in contradiction to that previously reported in the same vessel which relies on K^+^ channels (Löhn et al., 2002; Verlohren et al., 2004), this one depends on oxygen peroxide. The other mechanism is actually K^+^ channel-dependent but based on the opening of K^+^_Ca_, but not K^+^_ATP_, channels and, unlike the preliminary report on rat aortic PVAT, this one is endothelium-dependent (Gao et al., 2007).

The chemical nature of PVAT is still a matter of debate. Löhn and Dubrovska, by using specific inhibitors, discarded NO, prostaglandins and other mediators (Löhn et al., 2002; Dubrovska et al., 2004). Oxygen peroxide was the first substance proposed (Gao et al., 2007), followed by angiotensin 1-7 (Lee et al., 2009). By the same time, the gas hydrogen sulfide was suggested, in this case acting via K^+^_ATP_ channels (Fang et al., 2009). Subsequently, another research team showed that palmitate, acting through K^+^_V_ channels, might be the ADRF (Lee et al., 2011). Finally, adiponectin, which had been previously discarded as a candidate to ADRF (Fesus et al., 2007), was revisited in two papers published in 2013 demonstrating that adiponectin acts as an ADRF by eliciting anticontractile effects through the opening of K^+^_Ca_ channels (Lynch et al., 2013; Weston et al., 2013). One common feature in the quest for the identity of ADRF is K^+^ channel opening as well as smooth muscle hyperpolarization (Dubrovska et al., 2004). The acronym ADHF was proposed by Weston, who also coined the term EDHF (Weston et al., 1988) to refer to adipocyte-derived hyperpolarizing factors(s) (Weston et al., 2013).

### (b) Adipocyte-Derived Vasoactive Hormones

These are the well known vasoactive hormones such as angiotensin II, noradrenaline or NO. The novelty relies in that, interestingly, they can be generated by adipose tissue as well. So far, classic vasoactive substances that are, at the same time, adipokines are the following:

• NO. When generated by adipose tissue different from PVAT (Ribiere et al., 1996) and from PVAT (Dashwood et al., 2007).
• Prostacyclin. From general adipose tissue (Axelrod and Levine, 1981); from PVAT (Chang et al., 2012).
• Prostaglandin E_2_. From general adipose tissue (Shaw and Ramwell, 1968); from PVAT (Mendizabal et al., 2013; Ozen et al., 2013).
• TXA_2_. From general adipose tissue (Farb et al., 2014); from PVAT (Mendizabal et al., 2013; Meyer et al., 2013).
• Endothelin-1. From general adipose tissue (van Harmelen et al., 2008); from PVAT (Mendizabal et al., 2013).
• Angiotensin II. From general adipose tissue (Schling et al., 1999); from PVAT (Galvez-Prieto et al., 2008).
• Aldosterone. From general adipose tissue (Nguyen Dinh Cat et al., 2011); no report on PVAT.
• Noradrenaline. From general adipose tissue (Vargovic et al., 2011); from PVAT (Ayala-Lopez et al., 2014).

In some cases, such as that of prostaglandins, the adipocyte origin has been known for decades (Shaw and Ramwell, 1968), but only recently these have been found in PVAT (Chang et al., 2012; Mendizabal et al., 2013; Meyer et al., 2013).

One interesting issue is the ability of PVAT to release factors that are normally ascribed to endothelial cells. This has significant consequences, especially in obesity, in which hypertrophied visceral fat stores might produce some vasoconstrictors, like endothelin-1 or TXA_2_, the origin of which now we realize that is not only endothelial. Also, it is important to be aware of the fact that PVAT adipocytes are perfectly able to release angiotensin II or aldosterone (Engeli et al., 2000; Nguyen Dinh Cat et al., 2011) and add up to the regular kidney production.

Another “endothelium-like” property of PVAT is its ability to produce NO. This initially seemed to be conflicting with the well known vascular dysfunction of obesity. The conflict was solved by Gil-Ortega et al. (2010) who proposed that PVAT-derived NO, at least temporarily, plays an adaptive role in the early stages of obesity. Conflicting or not, we now know that PVAT is perfectly able to synthesize NO. It has been demonstrated that activation of β3 receptor in PVAT’s adipocytes by sympathetic nerve endings induce PKA-dependent NOS activation, thereby producing NO which elicits smooth muscle cell relaxation (Bussey et al., 2018). Furthermore, PVAT-derived NO appears to accompany the beneficial effects of physical exercise (Meziat et al., 2019).

It is known that acetylcholine, among other agonists, can stimulate adipocytic metabolism (Shaw and Ramwell, 1968). In this line, it has been proposed that acetylcholine stimulation of PVAT would be capable to produce NO and relax the surrounded artery, even if denuded of endothelium. (Xia et al., 2016). In this sense, PVAT would qualify as a real controller of vascular tone. This point has not been fully confirmed (Huang et al., 2010; Victorio et al., 2016) but it would mean that PVAT could support the endothelium, or even replace it in pathological conditions.

### (c) Energy Balance Adipokines

They are adipokines implicated in energy homeostasis. Therefore, production is altered in type II diabetes and in obesity and are involved in cardiovascular physiology and pathophysiology. These kind of adipokines comprises leptin, adiponectin, resistin, omentin, vaspin, visfatin and chemerin. Leptin receptors are present on endothelial cell membranes (Sierra-Honigmann et al., 1998). This means that vascular walls are potential targets of this hormone and posses the ability to generate NO (Frühbeck, 1999). Publications on the paracrine roles of leptin are contradictory. Initial information indicated that leptin causes NO-dependent (Kimura et al., 2000) or -independent (Nakagawa et al., 2002) vasodilation. This is not easy to fit with epidemiological research repetitively presenting leptin as related to the cardiovascular problems of obesity (Mattu and Randeva, 2013). Leptin released by PVAT elicits endothelial dysfunction in obese, but not lean, animals (Payne et al., 2010). However, many studies propose leptin as the ADRF acting via endothelial cells (Dashwood et al., 2011; Galvez-Prieto et al., 2012; Spradley et al., 2016). It is a paradox that leptin elicits vasodilation in an NO-dependent manner and simultaneously diminishes endothelium-dependent relaxations (Knudson et al., 2005).

Adiponectin is known as the adipokine that competes with leptin’s actions (Mattu and Randeva, 2013). Excessive release of the latter and scarcity of the former is related with the unwanted cardiovascular conditions occuring in obesity (Mattu and Randeva, 2013). Adiponectin has shown endothelial NO stimulating activity (Chen et al., 2003; Hattori et al., 2003) and NO-dependent vasodilating actions (Xi et al., 2005). Previous vasodilating evidences of adiponectin have led to the proposal that this adipokine might be the ADRF (Lynch et al., 2013; Weston et al., 2013).

Resistin, omentin and vaspin have not been investigated with regard to PVAT. However, it is known that resistin impairs endothelial function (Kougias et al., 2005), omentin elicits minor endothelium-dependent relaxations (Yamawaki et al., 2010) and vaspin improves endothelium-dependent relaxations elicited by acetylcholine (Kameshima et al., 2016). In contrast with these three, chemerin has been demonstrated to be generated by PVAT, with pro-contractile (Watts et al., 2013) as well as endothelium dysfunctioning (Neves et al., 2014) properties, acting possibly via NAD(P)H oxidase (Neves et al., 2015) and potentiating the effects of adrenergic nerve endings within PVAT (Darios et al., 2016). Visfatin, when obtained from PVAT, exhibits no effects on contractility (Wang et al., 2009) but, in non-PVAT set ups, visfatin has shown to cause an important impairment of endothelial function (Vallejo et al., 2011). Finally, chemerin has been identified as derived from PVAT. It has pro-contractile (Watts et al., 2013) as well as endothelium dysfunctioning (Neves et al., 2014) properties, acting possibly via NAD(P)H oxidase (Neves et al., 2015) and potentiating the effects of adrenergic nerve endings within PVAT (Darios et al., 2016).

### (d) Free Radicals

The opening publication connecting PVAT with free radicals was that already commented by Gao and coworkers in which oxygen peroxide acts as a vasodilator (Gao et al., 2007) and superoxide as a PVCF (Gao et al., 2006). It appears contradictory that reactive oxygen species (ROS) function as vasodilatory in some instances and vasoconstrictve in others. In this sense, a recent report highlights the dismutation of procontractile mitochondrial superoxide into anticontractile oxygen peroxide as a mechanism to reconcile the conflict between oxygen peroxide being, or not, relaxing (Costa et al., 2016). This superoxide-generating potential of PVAT is related to the anti-contractile properties of this tissue in obesity (Greenstein et al., 2009). Also to endothelial dysfunction in obesity as well (Ketonen et al., 2010). There is sufficient evidence to presume that NAD(P)H oxidase is the enzyme that synthesizes superoxide in PVAT (Gao et al., 2006; Virdis et al., 2015). Virdis et al. (2015) demonstrated the existence of a cause-effect association between TNFα and ROS production in PVAT. More recently, Costa and others linked their previously mentioned mitochondrial superoxide issue (Costa et al., 2016) to TNFα-induced oxidative stress (da Costa et al., 2017).

### (e) Adipocytokines

These are cytokines synthesized and released specifically by adipocytes (Trayhurn and Wood, 2004). Adipocytokines are produced during inflammation. They can activate inducible NOS (Amber et al., 1988) and, at the same time, impair endothelium-dependent relaxations (Aoki et al., 1989). PVAT adipocytes can release adipocytokines. Actually, PVAT is a fat depot with a demonstrated proinflammatory phenotype and releases more adipocytokines as compared with the subcutaneous depot (Chatterjee et al., 2009). This is true for the healthy non-obese individual (Chatterjee et al., 2009), but is aggravated in obesity (Henrichot et al., 2005). Aoki et al. (1989) published years ago a paper entitled “*Anti-EDRF effect of tumor necrosis factor*.” Based on the proinflammatory features of PVAT, it appears that this fat store produces anti-EDRF (anti-NO) substances. This has also been shown in myographic setups of arteries analyzed with or without PVAT (Payne et al., 2009) and specifically with the adipocytokine, TNFα (Donato et al., 2012). It must be highlighted that some reports disagree with the idea that PVAT is a harmful tissue merely because it exhibits a background release of adipocytokines. For example, some maintain that PVAT does not elicit endothelial dysfunction (Rittig et al., 2008) and numerous studies show that endothelium-dependent relaxations are perfectly normal in arteries with PVAT when weighed against those without PVAT (Ketonen et al., 2010; Mendizabal et al., 2013). Furthermore, a recent report indicates that the adipocytokine IL-6 seems to regulate the adipose beneficial changes associated to physical activity (Wedell-Neergaard et al., 2019). Endothelial dysfunction appears in vessels from obese animals (Ketonen et al., 2010; Mendizabal et al., 2013). The obese, consecutively, reveal lower anticontractile properties which can increase under a cytokine antagonist treatment (Greenstein et al., 2009).

Table 2 summarizes the substances released by PVAT specifying whether they are anti-contractile (relaxant) or pro-contractile. In some cases, the latter cause endothelial dysfunction of the surrounded vessel.

**Table 2:** Relaxing and contracting adipokines produced by PVAT.

Adipokine	Contracting (C) or relaxing (R) properties	References
Unknown	R	Löhn et al., 2002
Superoxide anion	C	Gao et al., 2006
Angiotensin 1-7	R	Lee et al., 2009
H_2_S	R	Fang et al., 2009
Angiotensin II	C	Lu et al., 2010
NO	R	Gil-Ortega et al., 2010
Leptin	C R	Payne et al., 2010 Galvez-Prieto et al., 2012
Visfatin^1^	C	Vallejo et al., 2011
Adiponectin	R	Lynch et al., 2013
Palmitate	R	Lee et al., 2011
Prostacyclin	R	Chang et al., 2012
PGE_2_	C R	Mendizabal et al., 2013 Ozen et al., 2013
TXA_2_	C	Mendizabal et al., 2013
Endothelin-1	C	Mendizabal et al., 2013
Chemerin	C	Watts et al., 2013
Noradrenaline	C	Ayala-Lopez et al., 2014
TNFα	C	Virdis et al., 2015
Vaspin^1^	R	Kameshima et al., 2016


*^1^Vessel not incubated with PVAT but with the adipokine.*

## New Pathways in the Regulation of Vascular Motion

Cells within the vascular environment keep surprising us for their abilities to interplay. The archetypal notion of the regulation of the vascular wall involving the classical mediators prostacyclin, NO, EDHF or EDCFs represented an outward communication. The advent of the always overlooked PVAT, turned over the whole concept into an inward signaling. It is worth to remember that an interaction of endothelial cells and adipocytes was proposed in the late eighties, before PVAT was known as such (Parker et al., 1989) and Richelsen published a review supporting the concept that adipocytes provide the endothelial cell with arachidonic acid for the synthesis of prostaglandins and that the release of these substances requires the action of both kind of cells (Richelsen, 1992). Since these initial proposals our understanding of the cross-talk between the different cells of the vascular milieu has experimented a remarkable growth. Some of these interactions can reach a high level of complexity, involving the nervous system or blood hormones and various types of vascular cells. The following are examples of these interactions:

1. Sympathetic nerve endings can, via PVAT adipocyte β3 receptors, activate adiponectin release which activates adiponectin receptors in endothelial cells that, in turn, elicit an AMP kinase-dependent NOS activation with NO release causing smooth muscle cell relaxation (Deng et al., 2010; Withers et al., 2014a).
2. Circulating atrial natriuretic peptide can activate PVAT adipocyte ANP receptors to cause PKG-dependent adiponectin release which follows the same route as above (Withers et al., 2014b).
3. Smooth muscle cell-derived ROS is released to the circulation as blood 4-hydroxynonenal. This, in the adipocyte, stimulates adiponectin release which inhibits NAD(P)H oxidase in endothelial cells, thereby protecting endothelial NO and, indirectly promoting the relaxation of the smooth muscle (Antonopoulos et al., 2015).

**FIGURE 4:**
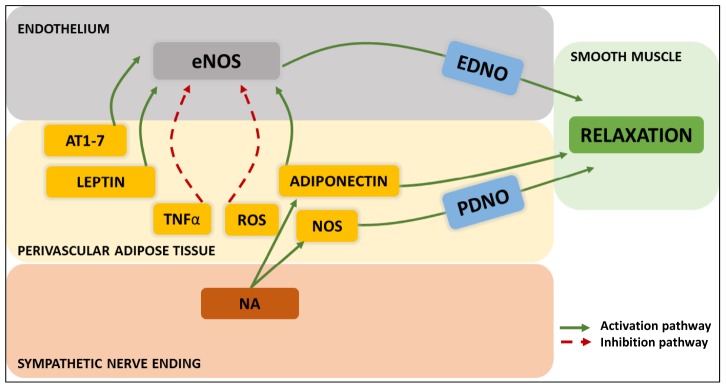
Modern patterns of “outside-to-inside” and “inside-to-outside” communication exhibited by PVAT adipocyte/sympathetic nerve endings-endothelial-smooth muscle cell- interactions. The three-way crosstalk so far discovered involves endothelium-derived NO (EDNO) on the luminal side, and on the perivascular side: angiotensin 1-7 (AT1-7), leptin, TNFα, reactive oxygen species (ROS), adiponectin, NO synthase (NOS), PVAT-derived NO (PDNO) and noradrenaline (NA) from the nerve endings.

An additional pathway involving the signaling of endothelial cells over PVAT adipocytes has also been investigated. Specifically, the potential interaction of endothelium-derived NO on the neighboring PVAT as compared to that on smooth muscle cells. This was however, not confirmed (Padilla et al., 2014). Finally, an unanticipated route of PVAT interactions with its immediate environment is the demonstration of a sensory nerve terminal-dependent (rather that sympathetic) activation of PVAT’s adipokine release with vasomotor consequences (Abu Bakar et al., 2017). Figure 4 summarizes the intercellular communication aimed to the control of smooth muscle tone.

## Author Contributions

EN compiled information and references and wrote the manuscript. SL assisted with reference managing, designed the figures, and assessed adequacy of the draft.

## Conflict of Interest Statement

The authors declare that the research was conducted in the absence of any commercial or financial relationships that could be construed as a potential conflict of interest.
